# Barriers, facilitators and preferences for the physical activity of school children. Rationale and methods of a mixed study

**DOI:** 10.1186/1471-2458-12-785

**Published:** 2012-09-14

**Authors:** María Martínez-Andrés, Úrsula García-López, Myriam Gutiérrez-Zornoza, Beatriz Rodríguez-Martín, María Jesús Pardo-Guijarro, Mairena Sánchez-López, Eugenio Cortés-Ramírez, Vicente Martínez-Vizcaíno

**Affiliations:** 1 Centro de Estudios Sociosanitarios, Universidad de Castilla-La Mancha, Cuenca, España; 2 Facultad de Trabajo Social, Universidad de Castilla-La Mancha, Cuenca, España; 3 Facultad de Terapia Ocupacional, Logopedia y Enfermería, Universidad de Castilla-La Mancha, Talavera de la Reina, Toledo, España; 4 Facultad de Educación, Universidad de Castilla- La Mancha, Cuenca, España; 5 Facultad de Educación, Universidad de Castilla-La Mancha, Ciudad Real, España; 6 Universidad de Castilla-La Mancha, Edificio Melchor Cano, Centro de Estudios Socio-Sanitarios, Santa Teresa Jornet s/n, Cuenca 16071, España

**Keywords:** Built environment, School, Obesity, Social environment, Physical activity, Mixed-method study, Focus groups, Barriers, Health behavior

## Abstract

**Background:**

Physical activity interventions in schools environment seem to have shown some effectiveness in the control of the current obesity epidemic in children. However the complexity of behaviors and the diversity of influences related to this problem suggest that we urgently need new lines of insight about how to support comprehensive population strategies of intervention. The aim of this study was to know the perceptions of the children from Cuenca, about their environmental barriers, facilitators and preferences for physical activity.

**Methods/Design:**

We used a mixed-method design by combining two qualitative methods (analysis of individual drawings and focus groups) together with the quantitative measurement of physical activity through accelerometers, in a theoretical sample of 121 children aged 9 and 11 years of schools in the province of Cuenca, Spain.

**Conclusions:**

Mixed-method study is an appropriate strategy to know the perceptions of children about barriers and facilitators for physical activity, using both qualitative methods for a deeply understanding of their points of view, and quantitative methods for triangulate the discourse of participants with empirical data. We consider that this is an innovative approach that could provide knowledges for the development of more effective interventions to prevent childhood overweight.

## Background

Despite the benefits of physical activity (PA), the proportion of children not meeting the current recommendations of PA is rapidly increasing in most of the developed countries, especially in the Mediterranean area [1,2], where the prevalence of overweight and obesity keeps growing at a very fast rate [3].

It is well known that low levels of PA tend to persist (tracking) from childhood to adolescence and adulthood [4-6]. In fact, PA levels usually decline through childhood and adolescence, being the peri-pubertal age a critical period of change [7]. School interventions in this age-group are thought as an applicable and effective way to improve PA levels, but consistent evidence about the optimal strategy to intervene is lacking [8].

Children typically perform PA under several situations, such as structured activities at school or sport facilities, and as unstructured ones like commuting to school or “active play”. Active play can be understood as the unstructured PA that takes place outdoor during children’s leisure time. In addition to physical health benefits, active play can also improve peer relationships and shows some other psychological benefits [9]. Understanding children’s perceptions about barriers and facilitators for both, structured and unstructured PA, could be helpful to design intervention and policy strategies to promote PA in this age-group.

### Built environment

Little is known about the influence of built environment on children’s physical activity [10-12]. The characteristics of the urban environment have been associated with various aspects of the population health [13]. The availability of infrastructures for leisure time, the climate of each area, distances, transport, the urban planning and configuration, and mainly access to workplaces and leisure areas, may have a deeply influence on the practice of habitual PA [6,14]. The urban environment in most of our modern cities does not facilitate the use of public spaces, reducing playground safety, and therefore influencing PA patterns of residents in those areas [15].

### School environment

School is the place where children are spending a great number of hours with opportunities for playing and with trained professionals who can encourage students to do PA [8,16,17]. As a result, the school environment is the most suitable place to intervention programs aimed to promote the PA by increasing the playtime of children [18]. Inside the school, during recess children can play freely with other children without inhibitions and therefore is an adequate setting for studying spontaneous PA [19,20].

### Social environment

The influences of social environment in the promotion of PA have received a lot of attention. Nevertheless, only a small number of studies have been focused on examining the influence of social and family relationships in the activities that children do during their leisure time [21]. Sharing time with classmates and/or relatives increases the active time of children and avoids sedentary behaviors [22-26]. Recent studies show a relationship between social environment, such as collective self-efficacy and social capital, and health outcomes like obesity [21].

The majority of studies aimed to analyze barriers and facilitators for PA in children have employed quantitative methods including neighborhood walkability scales to commuting or geographic information systems (GIS) [27]. Not many of these studies have examined the school environment and they have generally been focused on a quantitative measurement of sport facilities [18]. Likewise, there are not many studies involving qualitative research in order to know schoolchildren preferences on activities to be carried out on their free time, neither concerning children perception about their physical and social environment, and their relationship with PA [28]. To our knowledge very few of them have used mixed designs that combine qualitative methodology with objective measurement of PA [29,30].

There is a lack of insight about the reasons of the increasing prevalence of obesity in children from Spain and other Mediterranean countries [1,2]. This growing prevalence parallels the increasing frequency of sedentary behavior in children and adults, something difficult to understand if we considered the number of hours of day-light and good temperature enjoyed by these countries most of the year. Therefore, we think is important to know the children’s perceptions of their school, neighborhood and social environment, but also to validate this perception with objectively measured PA.

#### Rationale and objectives

This study is nested within “MOVI-2” project [31], that consists of two parts: 1) a cluster-randomized trial aimed to assess the effectiveness of a leisure time physical activity intervention (LTPA) in schools to reduce overweight/obesity and other cardiovascular risk factors, and 2) a mixed study aimed to know what are the children’s preferences to practice PA and how physical and social environment are influencing those preferences in a subsample of children.

The main objective of our study is to get a deep understanding of barriers, facilitators and preferences for PA of schoolchildren from Cuenca, Spain. To meet this goal we propose the following research questions:

 1) How children perceive the physical environment for the PA the school?

 2) How influence schoolchildren’s family and peers in the PA they do during leisure time?

 3) Which are the schoolchildren’s playground behaviors during recess time at the school?

 4) Is there any similarity between the children’s speeches about how they spent their leisure time and the PA they do?

## Design and methods

### Rationale for mixed design study

To get to know how barriers and facilitators for PA are perceived by schoolchildren we understood that qualitative methodology is the most appropriate approach, and within this methodology, the focus groups seems the most suitable technique to our objectives. Focus groups have proven to be an effective method to quickly gather a broad range of views and opinions about a given topic [32], especially in children [33]. However, in our opinion children aged 9-to-11 years have usually some difficulties in knowing their preferences and opinions about the issues of our study. Thus, we decided triangulate data from focal groups with another qualitative technique, drawings analyses, and with quantitative empirical measurement of the frequency and intensity of participant’s daily PA. Therefore, we can compare the amount of PA, objectively measured by movement sensors, with PA that children say they did in their speeches [19,34].

A phenomenological approach has been used to design and analyze the qualitative study, looking for a deep understanding of the motives and beliefs underlying the individual behaviors [35]. In our case, to get to know children preferences when performing PA and physical and social environment impact on those preferences. On the other hand, the participants wore accelerometers during seven consecutive days (including nights) in order to measure objectively the daily PA.

### Participants

Participants were a sub-sample of MOVI-2 study population, a cluster randomized trial (Clinicaltrials.gov number NCT01277224) carried out in 20 schools from the province of Cuenca, Spain, including 121 children aged 9-to-11 years, belonging to 4th and 5th grades of Primary Education (PE). Inclusion criteria are: both sexes, from different schools and places of origin, both urban and rural settings. The Clinical Research Ethics Committee of the Virgen de la Luz Hospital from Cuenca approved the study protocol. Talks were held with parents to inform them of the aims and methods of the study, and to request the signature of informed consent. We strongly encouraged them to take into account the views of children before signing this consent.

### Qualitative study

Two qualitative methods, complementary to each other, were used: the analysis of individual drawings of the physical environment [25] and focus groups [35]. The use of two techniques allowed us to gather not only more complete and reliable information, but also different levels of depth in the research questions [36]. Four preparatory sessions were used to test the adequacy of the methodology to the study objectives. As a result of these sessions we found that four-to-five children were an appropriate number of participants in each. For the drawings we considered that an individual drawing, but not a collective one, was the more efficient method to gather information about the children’s views of their neighborhood. We realized that drawings could be useful in focusing children’s thinking on the issues to be addressed later in the focus groups [29,30].

Therefore, after these preliminary pilot test, and given that individual drawings provided supplementary information, sessions began inviting participants to perform an individual drawing, and continue with a focus group including four-five both gender children. Each session was led by two researchers, one of them as moderator and the other one as observer, and was recorded on audio and video. From January to April 2011 were performed 26 sessions lasting on average 40 minutes each one. The process finished when the groups stopped offering new information, so we believed that information’s saturation level was reached [37].

#### Drawings

Participants were requested to draw a map that included the places where they used to go on weekdays and weekends during all the year. The use of drawings and maps helped to know perceptions of children about the physical space and the activities that were carried out [38]. For their performance, a paper sheet, a pencil and a rubber were facilitated, asking them to put the names of the different sites that were drawn 20 minutes time period was provided.

#### Focus groups

Since the volume of information could be very high, a topic guide (Table 1) was designed as script for the focus groups including the main questions and the principal probing questions. The topic guide was used as a script for the sessions, and was helpful to go closing themes (topics) where information was completed, and still deepening those in which we needed to know the opinion of the schoolchildren [35]. 

**Table 1:** Topic Guide for focus groups

**Main question**	**Probing questions**
What kind of activities do you practice?	Do you do them in the playground?
	Do you do them in your free time?
	Are these activities organized?
	Do you practice the same activities on weekdays and weekends?
	Do you like them?
What kind of games do you play?	Do you play them in the playground?
	Do you play them in your free time?
	Have you got video games console or computer?
	Do you watch TV?
Where do you practice these activities and games?	Are these places appropriately conditioned and safe? (Facilities and areas)
	How are these places?
	Green space, recreational space, leisure and sport facilities, home (kind of)
With whom do you practice these activities and games?	
When do you practice these activities and games?	
How do you move?	School, organized activities, shopping

#### Analysis

**Figure 1:**
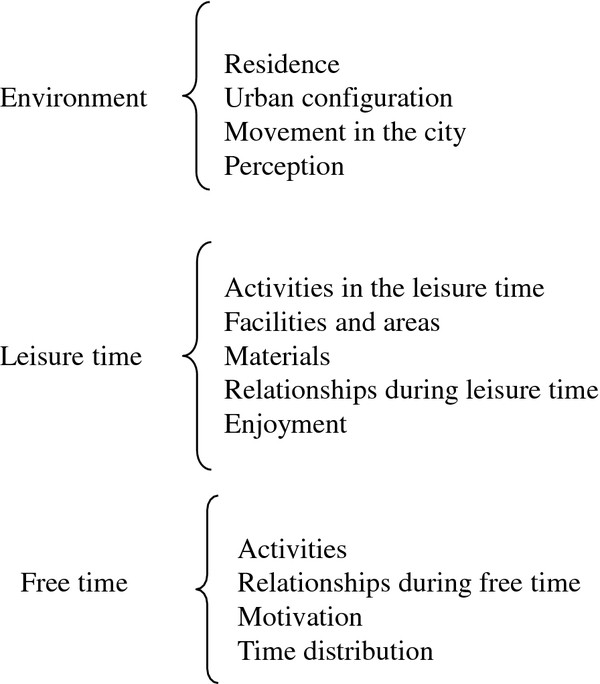
Coding Diagram.

After transcribing the focus groups, we arranged and organized texts. Three researchers expert in qualitative methodology (an anthropologist, a social worker and a political scientist) carried out, in a blinded way, the analysis of the transcripts. To be able to work with the information, the data were organized into three thematic blocks: “environment”, “recreation” and “free time”. These blocks were divided into codes in order to manage the information more efficiently and to identify different discursive positions (Figure 1).

The collection, analysis and interpretation of the data was carried out in an interactive process, so they reported to subsequent focus groups, allowing deepen into the key issues in which the information was not saturated or emerging issues that were not referred a priori by the topic guide [37].

We used content and discourse analysis of the focus groups transcripts. Following a first reading to understand concepts and opinions of the participants, a second reading was carried out, where text fragments were identified as belonging to each code. The associated ideas with each thematic area were extracted, and were developed in a framework where to explain each one of them. During this process, the specific concepts of the transcripts were identified in order to explain PA practice preferences, and those elements that had an important impact on those preferences. The concepts were tagged and organized in categories through open, axial and selective encoding processes. Once the individual analysis was implemented, research and hypothesis were shared among researchers, this helped a consensus reach on new categories and hypotheses and improve the understanding of the texts. Selective coding allowed central categories to be discovered, and the interrelation between the central categories and the rest was shown by an axial encoding.

The individual drawings were employed to assess the correspondence between these and the speeches of children involved. The frequency of places appearance and the importance of each type of place in the drawing (location, size and definition of it) were analyzed. In cases of discrepancy between speech and drawing of each participant, the discursive contributions of the speaker in the analysis of transcripts were not considered, assuming that the children was conditioned by the observer bias or by endorsement bias.

Software F4 was implemented to transcribe focus groups and the Atlas.ti 5.0 software was used for data processing of the data for the subsequent text analysis.

### Quantitative study: objective measurement of physical activity

Physical activity was measured using the GT1M accelerometer (ActiGraph TM, LLC, Fort Walton Beach, FL, USA). The students wore the accelerometer on the right hip for seven consecutive days. All participants were verbally instructed on how to use the accelerometer. The accelerometer was set to record PA data every minute (60s epoch). Sequences of 10 or more consecutive zero counts were considered non-wearing time and excluded from the analyses. Inclusion criteria were a minimum of four days of registration, including at least one weekend day and at least 600 registered minutes per day. Total activity average count per minutes (CPM) per day and total activity were measured differentiating between working days and weekend days. The minutes/day that were consumed in sedentary activity (0–100 CPM), light (101–2295 CPM), moderate (2296–4011 CPM) and vigorous (4012 - CPM) were calculated using the cut-points in Evenson [39]. Weekly recommendations of moderated/vigorous PA (MVPA) were valued if they were accomplished or not by the sum of the minutes per day of PA. Besides activity pattern was estimated in break and in spare time. Likewise, daily caloric expenditure was also estimated. The accelerometer data were analyzed with KineSoft software, version 3.3.2.0., and statistical analysis was carried out with the IBM SPSS 19 statistical software.

Along with the focus groups and the draws analysis, the accelerometer data were used to triangulate the information. Thus, we examine the correspondence among the speeches, the places appearing in the draw and the PA objectively registered. This triangulation allows us to control the possible biases.

## Discussion

As far as we know, this is the only Spanish study where qualitative methodology has been employed in order to meet physical and social environment perceptions as a barrier or facilitator of PA for children, from own children perspective; in the same way, only in another European study qualitative methodologies have been used in children to know the factors regarding facilitators of PA [9]. We believe it is an innovative analysis that could provide insight for the development of more effective interventions to prevent childhood overweight. In addition, the use of quantitative methods to quantify the frequency and intensity of PA allows triangulating this information to copy with observer and endorsement bias. Besides this method’s triangulation, we analyzed texts and draws with a double triangulation, three researches and three levels of coding (open, axial and selective) [36,40].

Potential limitations of this study are: 1) the age of the participants, 9-to-11 years, that in our opinion adds complexity to the design and analysis of focus groups [25]; 2) the current dominant culture of apparent promotion PA in schools and the mass media could mediate children’s discourse; and 3) the special characteristics of children’s movements, explosive but short-lived, could make that accelerometers, programmed in epochs of one minute so that the battery duration could have reached a record week, could not be registering less duration movement periods for children. Accelerometers are also known not to be a good instrument to record certain activities movements such as swimming or biking [41].

## Conclusion

In conclusion, despite the above limitations, our mixed design study seems to be a suitable approach to know the barriers and facilitators for the PA in schoolchildren.

## Abbreviations

PA: Physical activity; GIS: Geographic information system; LTPA: Leisure time physical activity intervention; PE: Primary education; CPM: Count per minute; MVP: Moderate/vigorous physical activity.

## Competing interests

The authors of this study declare that they have no competing interests.

## Authors’ contributions

All authors have contributed substantially to the manuscript. In particular, MMA, UGL and MGZ that were the principal investigators contributed to conception, design, analysis and interpretation of data. BRM, MJPG, MSL, ECR and VMV contributed to drafting of the manuscript and revising it critically. Also, VMV coordinated the study and reviewed the work done in the study. All authors provided final approval of the manuscript submitted.

## Pre-publication history

The pre-publication history for this paper can be accessed here:

http://www.biomedcentral.com/1471-2458/12/785/prepub
